# Uridine Depletion and Chemical Modification Increase *Cas9* mRNA Activity and Reduce Immunogenicity without HPLC Purification

**DOI:** 10.1016/j.omtn.2018.06.010

**Published:** 2018-06-30

**Authors:** Sriram Vaidyanathan, Krist T. Azizian, A.K.M. Ashiqul Haque, Jordana M. Henderson, Ayal Hendel, Sabrina Shore, Justin S. Antony, Richard I. Hogrefe, Michael S.D. Kormann, Matthew H. Porteus, Anton P. McCaffrey

**Affiliations:** 1 Department of Pediatrics, Stanford University, Stanford, CA, USA; 2 TriLink BioTechnologies, San Diego, CA, USA; 3 Department of Pediatrics I, Pediatric Infectiology and Immunology, Translational Genomics and Gene Therapy in Pediatrics, University of Tuebingen, Tuebingen, Germany; 4 The Mina and Everard Goodman Faculty of Life Sciences and Advanced Materials and Nanotechnology Institute, Bar-Ilan University, Ramat-Gan 52900, Israel

**Keywords:** mRNA, capping, Cas9, innate immunity, CRISPR, CleanCap, mRNA, uridine depletion, ARCA, Cap 1

## Abstract

The Cas9/guide RNA (Cas9/gRNA) system is commonly used for genome editing. mRNA expressing Cas9 can induce innate immune responses, reducing Cas9 expression. First-generation *Cas9* mRNAs were modified with pseudouridine and 5-methylcytosine to reduce innate immune responses. We combined four approaches to produce more active, less immunogenic second-generation *Cas9* mRNAs. First, we developed a novel co-transcriptional capping method yielding natural Cap 1. Second, we screened modified nucleotides in *Cas9* mRNA to identify novel modifications that increase Cas9 activity. Third, we depleted the mRNA of uridines to improve mRNA activity. Lastly, we tested high-performance liquid chromatography (HPLC) purification to remove double-stranded RNAs. The activity of these mRNAs was tested in cell lines and primary human CD34+ cells. Cytokines were measured in whole blood and mice. These approaches yielded more active and less immunogenic mRNA. Uridine depletion (UD) most impacted insertion or deletion (indel) activity. Specifically, 5-methoxyuridine UD induced indel frequencies as high as 88% (average ± SD = 79% ± 11%) and elicited minimal immune responses without needing HPLC purification. Our work suggests that uridine-depleted *Cas9* mRNA modified with 5-methoxyuridine (without HPLC purification) or pseudouridine may be optimal for the broad use of Cas9 both *in vitro* and *in vivo*.

## Graphical Abstract

**Figure:**
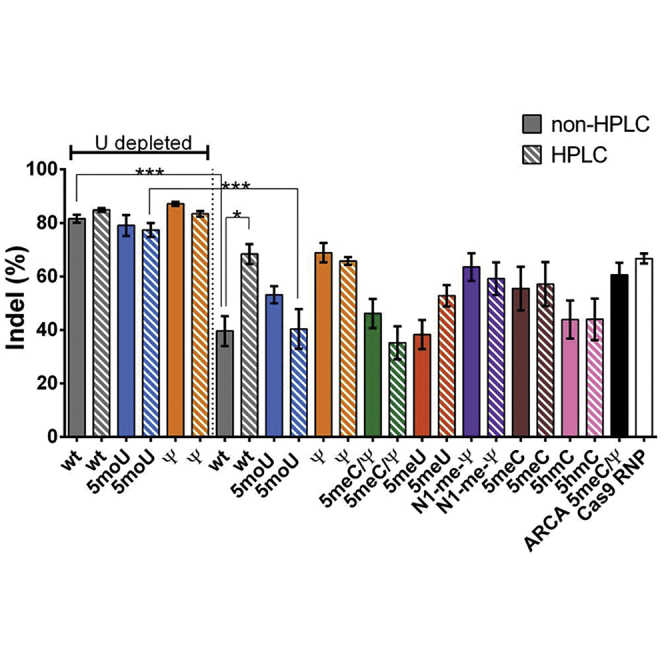

## Introduction

The Cas9/guide RNA (Cas9/gRNA) system, which is derived from the type II bacterial CRISPR adaptive immune system, is a powerful tool for manipulating genomes.^1^, ^2^, ^3^, ^4^ The Cas9/gRNA system consists of an RNA-guided nuclease (Cas9) and a single short gRNA. Upon delivery of these components to the nucleus of a cell, the guide strand directs the Cas9 protein to a specific chromosomal location, and Cas9/gRNA generates site-specific DNA double-strand breaks (DSBs), which are repaired by endogenous cellular mechanisms. Two major genome-editing events arise from the Cas9/RNA-induced DSBs: (1) a specific site can be mutated via mutagenic non-homologous end joining (NHEJ) by creating insertions or deletions (indels) at the site of the break, or (2) an exogenously introduced donor template can mediate a precise genomic sequence change via homologous recombination.^5^

Various methods have been described for delivery of the Cas9 protein into the nucleus. These include expression of Cas9 protein from a plasmid^6^ or viral vectors,^7^ transfection of recombinant Cas9 protein complexed to a gRNA (ribonucleoprotein or ribonucleoprotein [RNP] complex),^6^, ^8^, ^9^ or expression from a transfected *Cas9* mRNA.^6^ Expression of Cas9 protein from a plasmid or viral vector may be problematic because it risks integration of the promoter and/or *Cas9* gene cassette at the double-stranded break site, a feature of all double-stranded DNA vectors, or random integration of the DNA vector into the genome.^10^ By way of contrast, Cas9 protein and mRNA do not pose the risk of *Cas9* gene integration, and they also induce limited off-target effects due to transient expression.^5^, ^11^, ^12^ Although nanoparticle delivery of Cas9 protein has been reported, the most common approach to deliver transgenes into cells *in vivo* involves the use of mRNAs complexed with nanoparticles. This makes *Cas9* mRNA an attractive tool for genome editing in hard-to-transfect cells or tissues.

An ideal *Cas9* mRNA should mimic a fully processed mRNA and not activate innate immune pathways. Activation of these receptors induces inflammation, leads to translational inhibition, and causes mRNA degradation.^13^, ^14^, ^15^, ^16^ Our goal was to design and produce mRNAs that do not activate, or minimally activate, these RNA-sensing pathways. Exogenous mRNA can activate innate immunity pathways when various pattern recognition receptors (PRRs), present in both endosomes and the cytosol, recognize pathogen-associated molecular patterns (PAMPs) associated with exogenous RNA (viral or transfected RNA). Specifically, Toll-like receptors (TLRs) 7 and 8, which recognize single-stranded RNA (ssRNA),^17^, ^18^ and TLR3, which recognizes double-stranded RNA (dsRNA),^19^ must be avoided, since the activation of TLRs leads to inflammation and the inhibition of translation.^17^, ^18^, ^19^, ^20^ In addition, cytosolic PRRs to be avoided by *Cas9* mRNA include retinoic acid-inducible gene I (RIG-I), melanoma differentiation-associated protein 5 (MDA5), protein kinase R (PKR), and the interferon (IFN)-induced tetratricopeptide repeat (IFIT) proteins. RIG-I recognizes 5′ triphosphate (5′ppp)^21^, ^22^ or diphosphate,^23^ panhandle structures of viral genomic RNA (reviewed in Weber and Weber^24^), and uridine-rich sequences.^25^, ^26^, ^27^, ^28^ MDA5 is activated by binding very long dsRNA,^13^, ^29^ while PKR recognizes dsRNA stretches of at least 33 nt.^30^ Lastly, IFITs sense aberrant cap structures.^31^ Therefore, *Cas9* mRNA must avoid both endosomal TLRs and cytosolic PRRs to achieve maximal translation and protein activity. In this study, we tested the ability of cap structure modifications, chemical modifications, sequence engineering (uridine depletion), and high-performance liquid chromatography (HPLC) purification to reduce immune activity and increase *Cas9* mRNA activity.

## Results

### Eukaryotic Cap Structures

**Figure 1:**
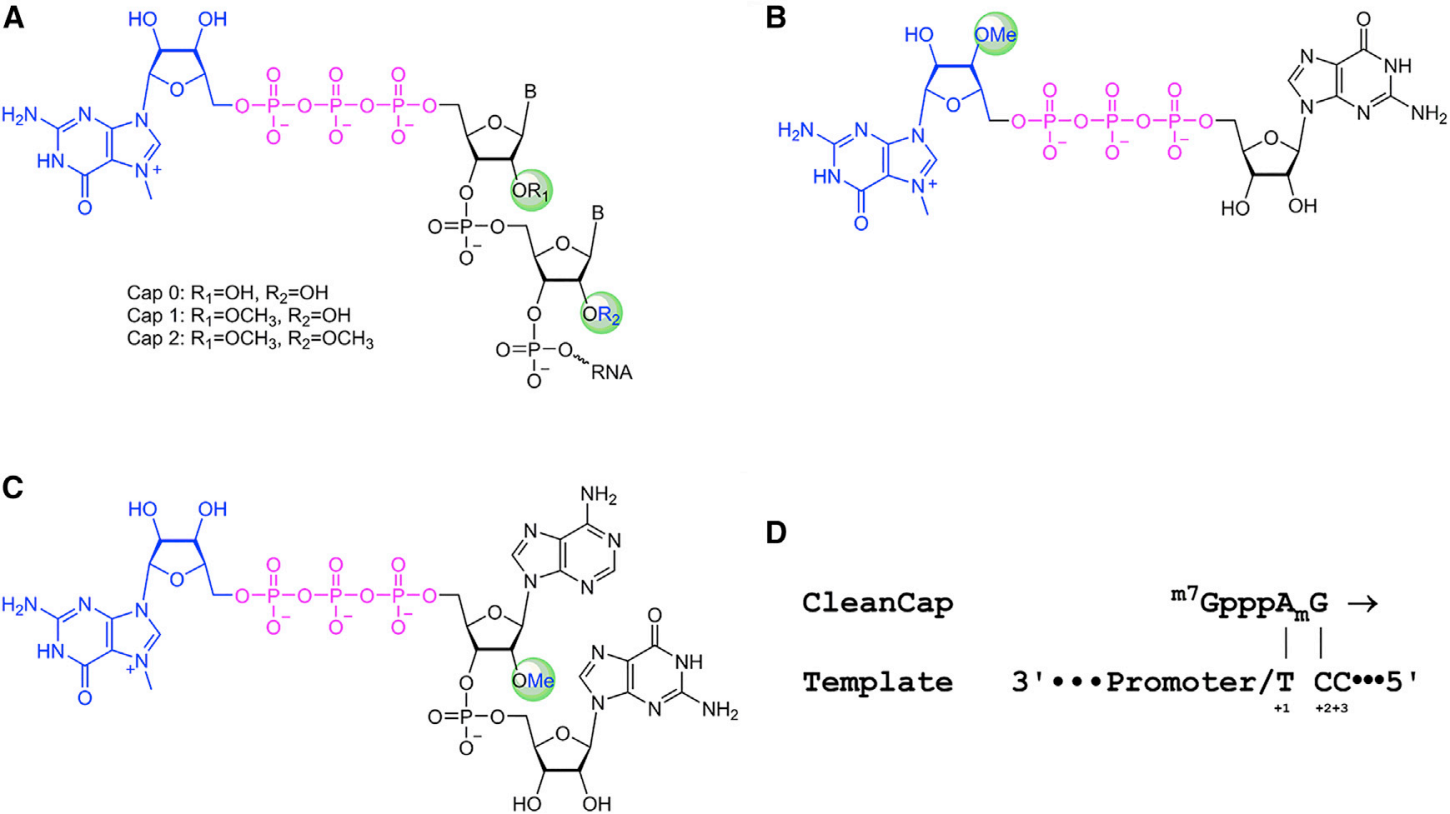
Eukaryotic Cap Structures and Cap Analogs (A) Eukaryotic cap structure. Presence of 2′-O-methyl groups at R_1_ and R_2_ determine if a cap structure is Cap 0, Cap 1, and Cap 2 as indicated. (B) Structure of anti-reverse cap analog used in standard co-transcriptional capping. (C) Structure of CleanCap AG Cap1 Trimer. (D) Proposed mechanism of CleanCap co-transcriptional initiation in which the AmG dimer portion of CleanCap docks onto the +1 and +2 template nucleotides. Initiation occurs when CleanCap couples to an NTP occupying the +3 position.

Eukaryotic RNAs are capped with a 7-methylguanosine (^m7^G) connected by a 5′-to-5′ triphosphate bridge to the first nucleotide. This structure is referred to as Cap 0. Cap 0 is important for the recruitment of translational initiation factors, and it prevents degradation of the mRNA. In higher eukaryotes, the 2′ ribose position of the first cap-proximal nucleotide is methylated to form a Cap 1 structure (^m7^GpppN_2′Om_ N), and, in ∼50% of transcripts, the second cap-proximal nucleotide is 2′ O-methylated to form Cap 2 (^m7^GpppN_2′Om_N_2′Om_) (Figure 1A).^32^

While the presence of Cap 1 and Cap 2 in eukaryotic RNAs has been known since the 1980s, the function of these modifications has remained largely unknown. Cytoplasmic viruses frequently possess mechanisms to acquire a Cap 1 structure (reviewed in Decroly et al.^33^ and Hyde et al.^34^). Many of these viruses are attenuated when their methyltransferases are inactivated, suggesting that cap structure may play an important role in self versus non-self-recognition. Cap 1 methylation has been shown to modulate binding or activation of innate immune sensors. For example, the binding affinity of IFIT-1 for Cap 1 and Cap 2 is much weaker than for 5′ triphosphate or Cap 0 RNAs, and IFIT-1 binding to non-2′-O methylated RNAs competes with the translational initiation factor EIF4E to prevent translation.^35^, ^36^ Cap 0 and 5′-triphosphate bind RIG-I with similar affinities, while Cap 1 modification abrogates RIG-I signaling.^37^ Similarly, Cap 1 prevents detection by MDA5.^38^

Frequently, synthetic mRNAs are co-transcriptionally capped by including a cap analog in excess in the transcription reaction. The current state of the art is co-transcriptional capping with anti-reverse cap analog (ARCA), which is a capped dimer with the chemical structure shown in Figure 1B. ARCA results in a Cap 0 mRNA. ARCA possesses a 3′-O-methyl group on the sugar adjacent to the ^m7^G, which prevents it from incorporating in the incorrect orientation, although this is not a naturally found Cap 0 modification. In general, T7 transcripts initiate at the +1-transcript position with guanosine triphosphate (GTP) incorporated opposite a +1-template cytosine. The ARCA cap analog is provided at a 4:1 excess over GTP in the transcription reaction such that, when competing with GTP, ARCA incorporates at the +1-transcript position ∼70% of the time leaving a Cap 0 structure (Figure 1A). Therefore, ∼30% of the time ARCA transcription initiates with GTP to yield an mRNA with a 5′ triphosphate. Our previously published work utilized an ARCA Cap 0 *Cas9* mRNA that was fully substituted with pseudouridine (Ψ) and 5-methylcytidine (5meC).^6^

Recently, we developed a co-transcriptional capping method called CleanCap that utilizes an initiating capped trimer instead of ARCA. Co-transcriptional capping with our CleanCap Cap1 AG trimer yields a naturally occurring Cap1 structure. In this study, we tested the ability of our newly developed CleanCap Cap1 AG trimer to improve *Cas9* mRNA activity or reduce its immunogenicity. We utilized T7 RNA polymerase to generate *in vitro*-transcribed mRNAs. The structure of a CleanCap Cap 1 AG trimer is shown in Figure 1C. With the CleanCap Cap 1 AG trimer, the +1 and +2 template nucleotides are thymidine and cytosine, respectively (Figure 1D). Our hypothesis is that the CleanCap Cap 1 AG trimer initiates by occupying the +1 and +2 transcript positions and elongation occurs when the CleanCap trimer couples to the nucleoside triphosphate (NTP) occupying the +3 position. We also tested an anti-reverse CleanCap trimers with a 3′-O-methyl group on the sugar of the ^m7^G (3′ O-methyl CleanCap AG) to prevent incorporation in the opposite orientation, but we found this to be unnecessary both in terms of yielding Cap1 structures and for indel formation (data not shown). A more extensive discussion of the CleanCap method will be presented elsewhere. To determine if we could improve on our previously published ARCA Cap 0 Ψ/5meC mRNA, we used CleanCap to generate a series of Cap 1 mRNAs that contained either wild-type (WT) bases or completely substituted with one or two modified bases (Table 1).

**Table 1:** List of *In Vitro*-Transcribed Modified *Cas9* Cap 1 mRNAs Made with CleanCap

Abbreviation	Full Name	Uridine Depleted	Cap Form	Screen
WT UD	wild-type bases	yes	Cap 1	full
5moU UD	5-methoxy uridine	yes	Cap 1	full
Ψ UD	pseudouridine	yes	Cap 1	full
WT	wild-type bases	no	Cap 1	full
5moU	5-methoxy uridine	no	Cap 1	full
Ψ	pseudouridine	no	Cap 1	full
5meC/Ψ	5-methyl cytidine/pseudouridine	no	Cap 1	full
5meU	5-methyl uridine	no	Cap 1	full
N1-me-Ψ	N1-methyl pseudouridine	no	Cap 1	full
5meC	5-methyl cytidine	no	Cap 1	full
5hmC	5-hydroxymethyl cytidine	no	Cap 1	full
N1-et-Ψ	N1-ethyl pseudouridine	no	Cap 1	initial
me1-Ψ /5meC	N1-methyl pseudouridine/5-methyl cytidine	no	Cap 1	initial
5moC	5-methoxy cytidine	no	Cap 1	initial
5camU	5-carboxy methyl ester uridine	no	Cap 1	initial
10% 5meC/2sU	5-methyl cytidine/2-thio uridine	no	Cap 1	initial
25% 5meC/2sU	5-methyl cytidine/2-thio uridine	no	Cap 1	initial
ARCA 5meC/Ψ	5-methyl cytidine/pseudouridine	no	Cap 0	initial

### Chemical Modification of mRNA

Although over 100 post-transcriptional modifications are found in RNA,^39^ only a subset are found in mRNAs. These mRNA modifications include N6-methyladenosine (m^6^A), inosine, N1-methyladenosine (m^1^A), Ψ, 5meC, and 5-hydroxymethylcytosine (5hmC).^40^, ^41^, ^42^ Such chemical modifications have been shown to reduce innate immune responses and improve mRNA activity.^43^, ^44^, ^45^

Karikó et al.^43^, ^44^, ^45^ demonstrated that substitution with modified bases reduced innate immune responses to transfected mRNAs. Based on this work, many first-generation mRNAs were modified with 5meC and Ψ.^46^ They showed that chemical modification of mRNA limited TLR signaling,^43^ decreased activation of 2′-5′-oligoadenylate synthetase,^45^ and decreased binding to PKR.^44^ Durbin et al.^47^ showed that RNAs modified with m^6^A bind RIG-I with reduced affinity, while Ψ, N1-methylpseudouridine (N1-me-Ψ), and 5meC RNAs bind RIG-I with high affinity yet fail to activate RIG-I signaling. Work by Peisley et al.^48^ also reported reduced RIG-I filament formation triggered by Ψ, 2-thiouridine (2sU), or m^6^A RNAs.

In addition to reducing innate immune responses, Karikó et al.^49^, ^50^ also showed that full substitution of mRNA with Ψ increased activity *in vivo*. Pardi et al.^51^ showed that N1-me-Ψ mRNAs were efficiently expressed in mice when delivered by a variety of routes. Andries et al.^52^ also reported that N1-me-Ψ mRNAs gave higher expression relative to Ψ-substituted mRNAs in mice.

Previously, we used partial substitution of mRNAs with 2sU and 5meC to express surfactant protein B (SP-B) to rescue SP-B-defective mice^53^ and to reduce asthma by expression of Foxp3.^54^ We have also used 2sU/5meC-modified mRNAs encoding zinc-finger nucleases and transcription activator-like effector nucleases (TALENs) in mouse lung, and we were able to demonstrate gene correction *in vivo* at the SP-B locus.^55^ In this study, we screened modifications such as Ψ, 5meC, N1-me-Ψ, 2sU, and others (Table 1) to identify modifications that improved Cas9 activity.

### Sequence Engineering

In addition to chemical modifications, studies have reported that sequence-engineered unmodified mRNAs may be superior to Ψ-modified RNAs *in vivo*.^56^, ^57^ Sequence engineering of mRNA utilizes the degeneracy of the genetic code to substitute specific nucleotides of an mRNA sequence or optimize codon utilization without altering the resulting amino acid composition. Several groups have reported that codon optimization could increase the activity of transfected mRNAs. Karikó et al.^50^ saw an increase in erythropoietin (Epo) expression in human dendritic cells upon codon optimization of both unmodified and Ψ-modified mRNA. Thess et al.^56^ sequence-engineered luciferase and Epo mRNAs by using only the most guanosine/cytosine (GC)-rich codons. They found that, while Ψ substitution improved the activity of non-sequence-engineered luciferase, Ψ modification decreased the expression of a sequence-engineered luciferase mRNA relative to an unmodified mRNA in HeLa cells. In mice, they found that sequence-engineered Epo performed better than non-sequence-engineered Epo mRNA and that Ψ modification of the optimized sequence decreased activity.

While designing our study, we found that, in the context of the luciferase open reading frame (ORF), depletion of uridines in the transcript using synonymous codons increased the luciferase activity for unmodified, Ψ, and 5-methoxyuridine (5moU)-modified RNAs (Figure S1). Based on these preliminary results and on reports by other groups that sequence engineering could improve mRNA activity,^56^, ^57^ we uridine-depleted the Cas9 ORF and synthesized 3 additional *Cas9* mRNAs containing WT bases, Ψ, or 5moU (Table 1). We selected these modifications for uridine depletion because work with other reporter mRNAs had shown improved activities with these modifications upon uridine depletion (data not shown). We compared the activity and immune response of these uridine depletion (UD) variants to the previously published ARCA Cap 0 Ψ/5meC mRNA.

### HPLC

dsRNA is produced as an undesired side product during *in vitro* transcription with T7 RNA polymerase.^58^, ^59^ This dsRNA could activate innate immune sensors, including TLRs, PKR, or MDA5. Karikó et al.^60^ reported that purification of mRNAs by HPLC reduced the levels of dsRNA impurities as assessed by a slot blot analysis with a dsRNA-specific antibody. They found that HPLC-purified Ψ-modified mRNAs had significantly higher activity *in vivo* than mRNAs that had not been HPLC purified.^60^ In this study, we adapted this method to HPLC purify a portion of each mRNA listed in Table 1 in order to test if HPLC purification of *Cas9* mRNA would reduce innate immune stimulation or increase indel activity.

Thus, we performed an unbiased investigation of ability of these different methods to increase *Cas9* mRNA activity with minimal immune activation. We found the highest frequency of genome editing with uridine-depleted 5moU, and these transcripts showed minimal *in vitro* and *in vivo* activation of the innate immune response without the need for HPLC purification.

### Uridine Depletion Improved Cas9 Activity by Indel Formation Assay

We conducted an initial indel formation screen of the mRNAs listed in Table 1 in primary CD34^+^ hematopoietic stem and progenitor cells (HSPCs) mobilized from peripheral blood. We used primary human cells for these assays as they are more sensitive to the immunostimulatory activity of delivered nucleic acids than cancer cell lines. Cells were co-transfected with 3 μg *Cas9* mRNA and 2 μg MS-single-guide RNA targeting the interleukin-2 receptor subunit gamma (IL2RG) locus. As a control, we also included Cas9 RNP using 6 μg Cas9 protein with 3.2 μg guide strand, as we have previously described.^6^ Indels were quantitated using TIDE analysis.^61^ Based on this initial screen, we narrowed our list to 11 candidate mRNAs (each with and without HPLC purification; Table 1, lead candidates). We tested the lead candidates on CD34^+^ cells from five different donors.

**Figure 2:**
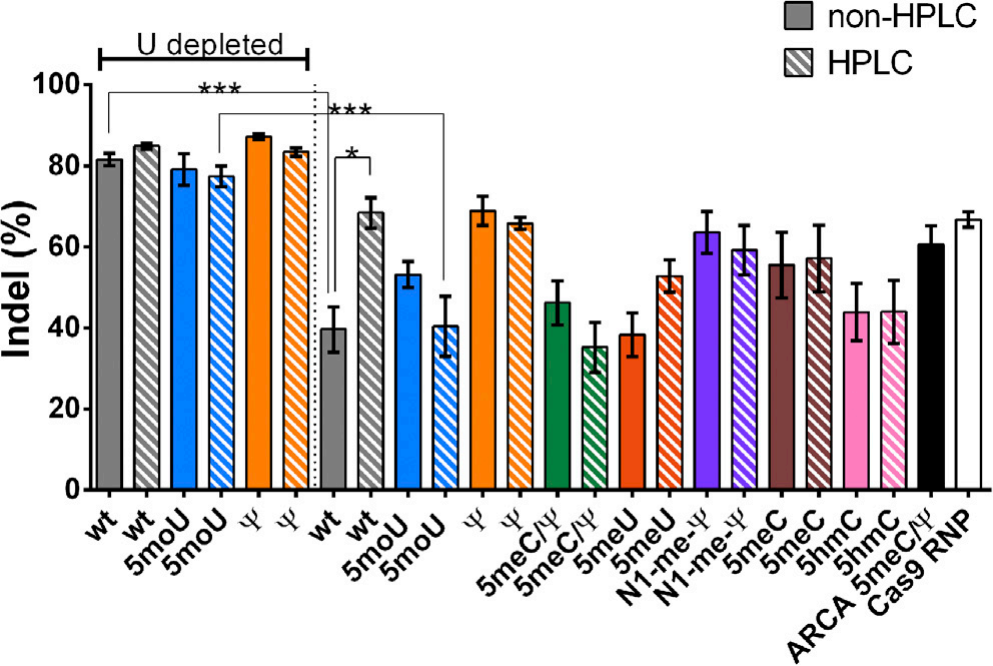
Indel Formation in CD34+ HSPCs Nucleofected with Modified *Cas9* mRNAs CD34+ HSPCs were nucleofected with 3 μg of the indicated *Cas9* mRNA and 2 μg IL2RGlocus MS-sgRNA. 6 μg Cas9 RNP complexed to 3.2 μg IL2RGlocus MS-sgRNA was nucleofected for comparison. ARCA 5meC/Ψ is our previously published *Cas9* mRNA^6^ and was also included for comparison. Cells were isolated after 4 days, and indel formation was assessed by TIDE analysis. Bars represent mean ± SEM of at least 5 independent transfections. White and gray bars indicate RNeasy and HPLC-purified mRNAs, respectively. sgRNA complexed to Cas9 RNP was included as a control. ***p < 0.0005 and *p < 0.05.

The uridine-depleted mRNAs (WT UD, WT UD HPLC, 5moU UD, 5moU UD HPLC, Ψ UD, and Ψ UD HPLC) yielded the highest indel rates (∼77%–87%) (Figure 2). This was a major improvement over our first-generation Cap 0 5meC/Ψ mRNA, which gave 61% indel. Indel frequencies with the Cas9 RNP complexed to guide were 67%. WT UD and 5moU UD HPLC showed a statistically significant improvement in indel frequency relative to their non-uridine-depleted counterparts (p < 0.0001). Among the non-uridine-depleted sequences, WT HPLC, Ψ, and Ψ HPLC gave 66%–69% indel formation. The chemical modifications in combination with UD did not improve *Cas9* mRNA activity. Even among the non-UD samples, the chemical modifications did not outperform WT HPLC-purified samples. Chemical modifications among non-UD samples were still relevant from the perspective of immune response, as discussed below.

To our surprise, in most cases, HPLC purification did not increase indel formation significantly. One notable exception was WT, where HPLC purification improved activity from 40% to 68% (p = 0.028). To assess the level of dsRNA contamination and depletion of dsRNA during HPLC, we adapted a previously described dsRNA immunoblot with a dsRNA-specific antibody.^60^ Based on this qualitative assay, we estimate that HPLC purification reduced dsRNA levels by 50%–80%, while uridine depletion reduced dsRNA levels by approximately 30% (Figure S2). While Karikó et al.^60^ interpreted a decrease in blot signal to reflect removal of dsRNA, another alternative is that heating and denaturation of the mRNA during HPLC unfolded long double-stranded intramolecular structures within the purified mRNA, resulting in a decrease in slot blot signal.These experiments suggest that uridine depletion of *Cas9* mRNA can significantly improve CRISPR gene editing and may then obviate the need for HPLC purification in CD34+ cells.

Lastly, we tested the influence of the CleanCap, ARCA, and anti-reverse CleanCap cap structures on the performance of the 5moU UD mRNA. Notably, there was no significant difference in the indel activity between the 5moU UD ARCA CleanCap and 3′ O-methyl CleanCap *Cas9* mRNA (Figure S3).

### IFN Responses in Differentiated THP-1 Cells Transfected with Unmodified and Modified mRNAs

**Figure 3:**
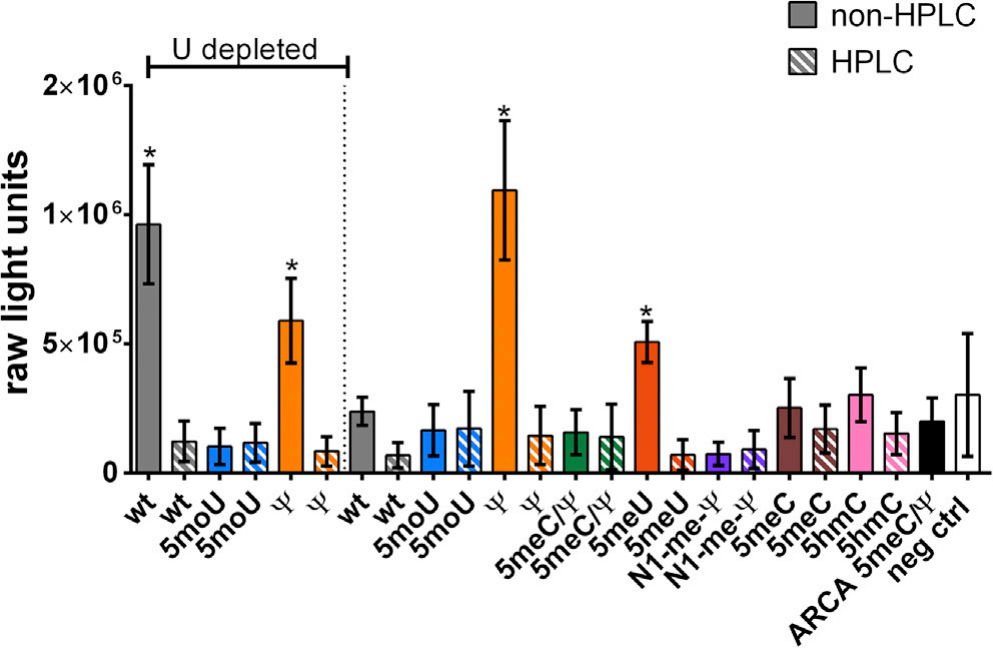
IFN Response Generated by THP-1 Dual Cells Transfected with Modified *Cas9* mRNAs THP-1 dual cells were transfected in sextuplicate with 100 ng of the indicated mRNAs complexed with 1 μL transfection reagent mRNA-In. At 24 hr, Lucia expression in the media was assayed as a measure of IFN activity. Bars represent mean ± SEM of three independent assays comprising a total of 18 replicates. *p < 0.05.

We next tried to narrow down mRNA variants with improved activity that also induced low innate immune responses in the *ex vivo* setting. We used an IFN reporter cell line to assess IFN stimulation upon transfection of the various mRNAs into THP-1 Dual cells. THP-1 Dual cells are human monocyte stable transfectants, which, upon IFN stimulation, express a secreted coelenterazine luciferase (Lucia) driven by the ISG54 (IFN-stimulated gene) minimal promoter and five IFN response elements. The majority of modified mRNAs did not induce significant IFN responses above the negative control with or without HPLC purification (Figure 3). Notably, Ψ-modified mRNAs induced elevated IFN responses both with and without UD. Apart from Ψ-modified mRNA, WT UD and 5meU that had not been HPLC purified gave significantly elevated IFN signaling relative to the negative control. In each case, HPLC purification reduced IFN signaling to background levels. To our surprise, non-HPLC-purified WT *Cas9* did not induce significant IFN signaling. Among the UD variants, 5moU UD did not exhibit significant IFN response even without HPLC purification, and, thus, it appears to be an attractive choice for editing applications.

### IL-12, TNF-α, and IL-6 Measurements in Whole Blood

**Figure 4:**
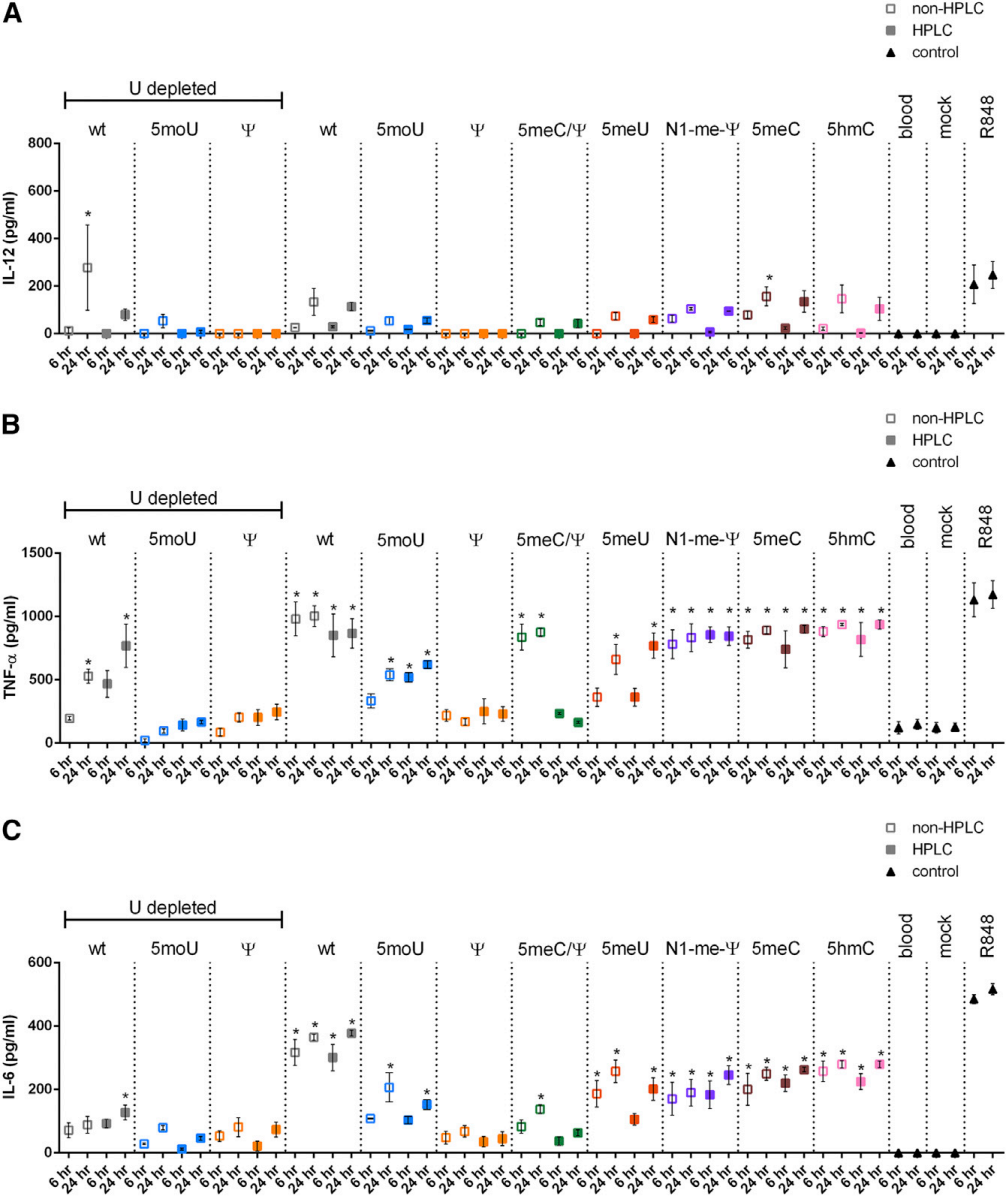
Amounts of IL-12, TNF-α, and IL-6 in Whole Human Blood Transfected with Modified *Cas9* mRNAs To assess immune responses to transfected mRNAs, whole blood from healthy human volunteers (n = 3) was transfected with 10 μg mRNA complexed with 10 μL TransIT (https://www.mirusbio.com/). After 6 or 24 hr of incubation, sera were isolated and (A) IL-12, (B) TNF-α, or (C) IL-6 was measured by ELISA. Bars represent mean ± SEM. *p < 0.05 relative to 6-hr blood-only control.

In addition to *ex vivo* editing of isolated cells, the Cas9/gRNA system can be applied in whole organisms. The activation of the innate immune response *in vivo* has been a major barrier to gene therapy vectors, even inducing a patient death.^62^ We next sought to assess immune responses in the more complex environment of whole human blood and identify mRNA variants that induced lower immune responses in whole blood. Whole blood obtained from healthy donors was transfected with mRNAs that were complexed with TransIT delivery reagent. At 6 and 24 hr, serum was isolated and interleukin (IL)-12 (Figure 4A), tumor necrosis factor alpha (TNF-α) (Figure 4B), and IL-6 (Figure 4C) were measured by ELISA.

Most *Cas9* mRNA variants did not induce IL-12 secretion. Among the UD samples, levels of IL-12 were significantly elevated relative to blood-only controls for WT UD, but not the other variants, at 6 and 24 hr. For the non-UD variants, levels of IL-12 were significantly elevated relative to 6-hr blood-only control for 5meC at 24 hr. While some other groups were slightly elevated, they did not reach statistical significance.

Many *Cas9* mRNA variants induced TNF-α secretion. Among the UD samples, TNF-α levels reached significance at 24 hr only for WT UD and WT UD purified by HPLC. Among the non-UD samples, TNF-α levels were significantly elevated at 6 and 24 hr regardless of HPLC purification for WT, 5moU, N1-me-Ψ, 5meC, 5meC/Ψ, and 5hmC. But HPLC purification abolished TNF-α induction only for 5meC/Ψ. Interestingly, 5moU UD did not increase TNF-α even though the non-UD 5moU increased TNF-α.

Most *Cas9* mRNA variants that induced TNF-α secretion also induced IL-6 secretion (except WT UD). For uridine-depleted sequences, IL-6 levels reached significance only for WT UD purified by HPLC at 24 hr. Among the non-UD samples, IL-6 levels were significantly elevated at 6 and 24 hr regardless of HPLC purification for WT, N1-me-Ψ, 5meC, and 5hmC. 5meU increased IL-6 at 6 and 24 hr without HPLC purification but only increased IL-6 after 24 hr when purified by HPLC. For 5meC/Ψ, IL-6 levels were significantly elevated at 24 hr, but HPLC purification abolished IL-6 induction. IFN-α was also tested, but it was not measurably induced by any of the tested mRNAs (data not shown).

### IL-12, TNF-α, and IL-6 Measurements in Mice

**Figure 5:**
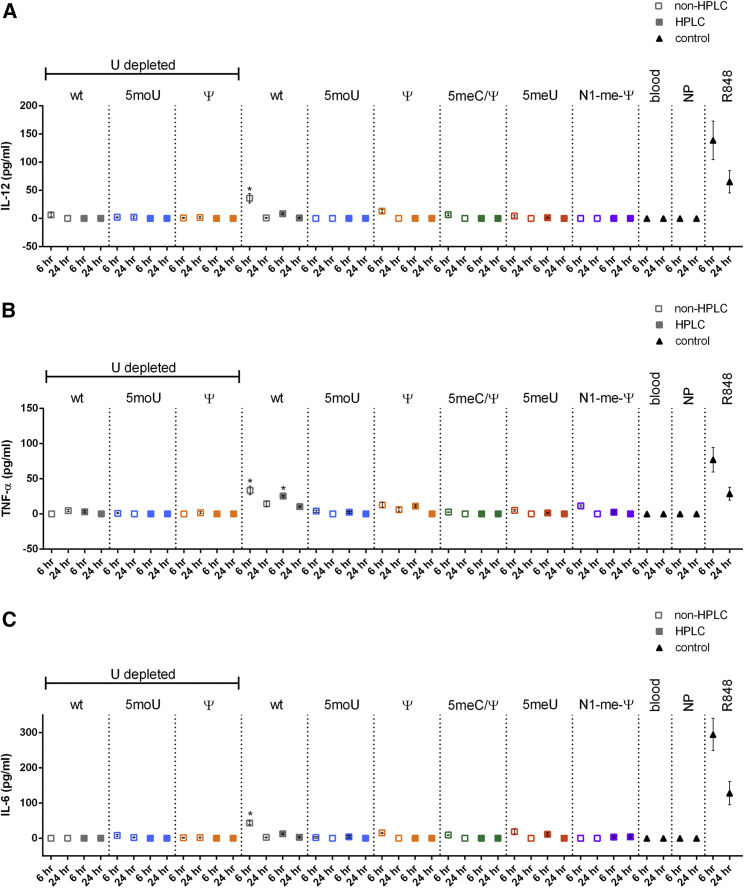
Amounts of IL-12, TNF-α, and IL-6 in the Sera of Mice after Intravenous Infusion of Modified *Cas9* mRNAs To assess immune responses *in vivo*, 20 μg *Cas9* mRNA encapsulated in chitosan-coated PLGA nanoparticles was injected intravenously (n = 3) into the tail vein of mice. After 6 or 24 hr of incubation, sera were isolated and (A) IL-12, (B) TNF-α, or (C) IL-6 was measured by ELISA. Blood treated with R-848 serves as a positive control. Bars represent mean ± SEM. *p < 0.05 relative to 6-hr blood-only control.

To measure immune responses in mice *in vivo*, *Cas9* mRNAs were encapsulated in chitosan-coated poly-D,L-lactide-co-glycolide (PLGA) nanoparticles and injected into the tail vein of mice (n = 3). At 6 or 24 hr, mice were sacrificed and serum IL-12 (Figure 5A), TNF-α (Figure 5B), and IL-6 (Figure 5C) were measured by ELISA. None of the UD samples showed elevated serum IL-12, TNF-α, or IL-6. Only WT non-HPLC-purified IL-12 and IL-6 levels were significantly elevated relative to 6-hr blood-only control at 6 hr. Similarly, at 6 hr, TNF-α levels were elevated relative to control for both WT and WT HPLC, while no other significant changes were observed.

## Discussion

### Improvement in *Cas9* mRNA Activity by UD

In our current study, we applied four design principles to improve the activity of our previously published ARCA Cap 0 Ψ/5meC *Cas9* mRNA.^6^ These included exploring sequence engineering, screening different modified bases, examining the influence of HPLC purification, and using a Cap 1 structure. Among these strategies, a combination of UD with 5moU modification was able to achieve indel rates as high as 87% in primary CD34^+^ HSPCs while avoiding immune responses even in the absence of HPLC purification (Figure 2).

UD was most effective in increasing *Cas9* mRNA activity, but further chemical modification was necessary to reduce immunogenicity. However, this increase in indels by UD variants only reached statistical significance for WT UD and 5moU UD HPLC relative to their non-uridine-depleted counterparts (p < 0.05). We also tested the off-target activity of 5 moU UD against ARCA 5meC/Ψ at a previously reported off-target site (chromosome [chr]1: 167730172–167730194).^6^ The off-target indel activity was close to the limit of detection, and it was not significantly different between 5 moU UD (8% ± 1%) and ARCA 5meC/Ψ (5% ± 2%). Uridine depletion may improve indel levels by increasing protein expression, reducing immune responses, or a combination of the two effects. In our studies with luciferase (Figure S1), uridine depletion increased protein expression, but the percent increase was significantly different between 5moU and Ψ. Consistent with our results, studies have reported that codon optimization can influence both expression and mRNA stability.^63^ More specifically, GC-enriched (adenosine/uridine [A/U]-depleted) genes have been reported to exhibit higher steady-state mRNA levels when expressed using plasmids.^64^ The study further seemed to suggest that UD did not affect mRNA degradation rates, and it speculated that some mRNA-processing pathway(s) may influence mRNA levels.^64^ It is also possible that uridine depletion could reduce recognition by TLRs. Interestingly, it has been reported that TLR7 recognizes uridine stretches.^20^ Tanji et al.^65^ also reported that uridine containing single-stranded RNA degradation products could be sensed by TLR8. Further studies with a variety of primary sequences may be necessary to understand the mechanism by which the combination of mRNA sequence and chemical modifications influences protein translation in different cell types.

### Immunogenicity of UD and non-UD *Cas9* mRNA Variants

To test immune responses, we employed two complementary assays, a whole-blood assay and an *in vivo* mouse model. The whole-blood assay appears to be the most sensitive assay for monitoring these responses. The difference between the whole-blood and *in vivo* results may reflect difficulties in measuring systemic cytokines in mice in response to local delivery to a subset of cells. In these assays, uridine depletion also decreased most, but not all, immune responses elicited by WT, 5moU, and Ψ. Specifically, UD reduced TNF-α and IL-6 for 5moU (Figures 4B and 4C). For WT *Cas9* mRNA, UD significantly reduced IL-6 levels in whole-blood assay (Figure 4C) and reduced TNF- α, IL-6, and IL-12 in the mouse assay (Figure 5). However, UD was insufficient to reduce IFN responses completely for Ψ and increased IFN for WT (Figure 3).

The mechanism by which uridine depletion affects immune responses is unclear. It is possible that sequence engineering could influence binding to PRRs and thereby minimize the translational inhibitory effects that are activated through PRRs. It seems possible that PRRs designed to recognize aberrant RNAs might focus on uridine residues, as this is a major difference between DNA and RNA. For example, it was reported that triplets of sequential uridines could activate TLR-7 and cause dendritic cells to release IFN-α.^20^ Likewise, RIG-I is reported to recognize uridine-rich sequences.^25^, ^26^, ^27^, ^28^ Further research will be required to define the precise mechanism by which uridine depletion improves the activity of some *Cas9* mRNA variants despite immune activation.

In the context of nucleofection of CD34^+^ HSPCs, we show that *Cas9* WT UD can have similar indel formation rates as Ψ and 5moU-modified RNAs (Figure 2). Thus, at least in CD34+ cells, uridine depletion may be sufficient and chemical modification may not be necessary. By way of contrast, reducing immune responses may be still important for *in vivo* applications, since the route of delivery may be an important determinant of efficacy and innate immune induction.^66^ Since electroporation likely bypasses the endosomal compartment (where RNA-sensing TLRs reside), it remains to be determined if the same results would be observed with lipid or polymer transfection. A significant body of research supports the idea that chemical modification of mRNA can improve its activity by reducing innate immune stimulation in several instances.^49^, ^50^, ^51^, ^52^, ^53^, ^54^, ^55^, ^60^ Indeed, chemical modification of mRNA has been reported to decrease binding to or activation of TLRs,^43^ 2′-5′-oligoadenylate synthetase,^45^ PKR,^44^ and RIG-I.^47^, ^48^ Chemical modifications are known to change the structure, base pairing, and codon/anti-codon pairing of mRNAs (reviewed in Harcourt et al.^67^), and they may thus make them poor substrates for PRRs. In contrast to the above reports, several groups have reported that sequence-engineered mRNAs may not require chemical modification. Thess et al.^56^ found that, while chemical modification improved the activity of some sequences, when they used mostly GC-rich codons, WT mRNAs had the highest activity. Kaufmann et al.^57^ found that when they formulated mRNAs in lipid nanoparticles and delivered them intravenously to mice, WT and Ψ mRNAs had similar activities and immune responses. Thus, the need for chemical modification may depend on the individual mRNA sequence as well as the route of administration. Since UD did not completely inhibit immune responses, we also explored the combination of UD with chemical modification.

In our studies, most chemical modifications reduced IFN responses except for Ψ and 5 meU. This suggests that the chemical modification present in many of the *Cas9* mRNAs tested may mask dsRNA recognition. It is also possible that these modifications are not recognized by the relevant PRRs. The observation that Ψ did not reduce IFN stimulation was somewhat surprising based on the literature.^49^ Unfortunately, all chemically modified variants, except Ψ, still induced TNF-α and IL-6 in the absence of UD (Figures 4 and 5). Therefore, we tested the ability of HPLC to reduce the remaining immune responses.

### Influence of HPLC Purification on Reducing Immunogenicity of *Cas9* mRNA Variants

HPLC was successful in reducing IFN responses when a combination of chemical modification and UD did not reduce IFN (WT UD, Ψ UD, Ψ, and 5meU) (Figure 3). Surprisingly, this reduction in immune response by HPLC purification only resulted in significant improvement in indel frequencies for non-uridine-depleted WT (Figure 2). This may reflect that the natural substrate for dsRNA-sensing PRRs is WT RNA. The reduction of IFN response after HPLC purification is consistent with literature (Karikó et al.^60^), especially for Ψ.^60^ However, we also noticed that several chemical modifications (for 5moU UD, 5moU, 5meC/Ψ, N1-meΨ, 5meC, and 5hmC) showed no IFN response even without HPLC purification. Indeed, on further comparison with Karikó et al.,^60^ we noticed that the influence of chemical modification on IFN and TNF responses was protein dependent in their study. Thus, our data are broadly consistent with Karikó et al.^60^ that HPLC purification reduces IFN responses if they are present.

In contrast to IFN responses, HPLC was unsuccessful in reducing TNF-α and IL-6 secretion induced by all variants except 5meC/Ψ, as measured by the whole-blood assay. This result is inconsistent with Karikó et al.^60^ Unlike Karikó et al.,^60^ our results show that we were only able to deplete, but not completely rid, our mRNAs of dsRNA using HPLC purification (Figure S2). Thus, it is also possible that the remaining dsRNA in the HPLC-purified samples is sufficient to trigger immune responses that are equivalent to the non-HPLC-purified RNAs, rendering HPLC purification insufficient to provide a benefit within the context of CD34^+^ HSPCs. These results suggest that a further reduction in dsRNA may be necessary to reduce IL-12, IL-6, and TNF- α and increase indels.

### Influence of Capping Strategy *Cas9* mRNA Activity and Immunogenicity

Lastly, the capping strategy did not seem to impact indel formation (Figure S2). We had hypothesized that the presence of Cap 1 in our mRNAs may also decrease binding to PRRs and reduce the need for HPLC purification. It has been reported that MDA5 does not recognize Cap 1 mRNAs efficiently.^38^ It remains to be tested if other dsRNA sensors such as RIG-I have decreased sensitivities to Cap 1 mRNA. We only compared the Cap 0 and Cap 1 modifications for the 5moU UD variant mRNAs, and we did not observe any difference in indel levels (Figure S3). However, there was a small but statistically significant increase in IL-12 production, but not TNF-α or IL-6, in the whole-blood assay in response to Cap 0 ARCA relative to the Cap 1 CleanCap analogs (Figure S4). There was no difference in cytokine production in the less sensitive mouse model system for the Cap 0 or Cap 1 mRNAs (Figure S5). It is possible a Cap 1 structure would have provided more benefit for 5moU-modified mRNA in the absence of UD.

### Conclusions

In summary, we have used a variety of approaches in combination to identify several improved *Cas9* mRNAs. Among these strategies, uridine depletion resulted in the greatest increase in indel levels (Table 2), but WT UD samples still elicited innate immune responses. Ψ UD showed high indel levels and reduced both IL-6 and TNF-α, but not IFN. IFN induction by Ψ UD was resolved by using HPLC. By way of contrast, 5moU modification of WT UD *Cas9* mRNA maintained indel frequencies and reduced all immune responses, even without HPLC purification. Given that no benefit was seen upon HPLC purification, HPLC would not be recommended for this modification, because of the additional cost and the significant loss of yield upon HPLC purification. Taken together, 5moU UD would be the preferred candidate for *Cas9* mRNA given that it does not require HPLC purification (Table 2). Future studies may be necessary to investigate the influence of sequence engineering and chemical modification on the *in vivo* activity of 5moU *Cas9* mRNA.

**Table 2:** Summary of Assay Results for Uridine-Depleted Sequences

	WT UD	WT UD HPLC	5moU UD	5moU UD HPLC	Ψ UD	Ψ UD HPLC
High indel	+	+	+	+	+	+
Lack of IFN in THP-1 Dual cells	–	+	+	+	–	+
Lack of IL-12 in whole blood	–	+	+	+	+	+
Lack of TNF-α in whole blood	–	–	+	+	+	+
Lack of IL-12 *in vivo*	+	+	+	+	+	+
Lack of TNF-α *in vivo*	+	+	+	+	+	+

## Materials and Methods

### Transcription Templates and Sequence Optimization

The Cas9 ORF with C-terminal nucleoplasmin nuclear localization signal (NLS) and hemagglutinin epitope tag was provided by Feng Zhang. It was cloned into the mRNA expression vector pmRNA, which contains a T7 RNA polymerase promoter, an unstructured synthetic 5′ UTR, a multiple cloning site, and a 3′ UTR that was derived from the mouse α-globin 3′ gene. An N-terminal SV40 NLS was added to generate the vector pmRNA_NLS_Cas9_NLS. A transcription template was generated by PCR using mRNA forward primer 5′-TCGAGCTCGGTACCTAATACGACTCAC-3′ and mRNA reverse primer T(2′OMe)T(2′OMe)(T)_118_CTTCCTACTCAGGCTTTATTCAAAGACCA-3′. The poly A tail was encoded in the template, and the resulting PCR product encoded a 120-nt poly A tail.The uridine-depleted plasmid pmRNA_UD_NLS_Cas9_NLS was created by codon optimization of the *Cas9* mRNA ORF contained within pmRNA_NLS_Cas9_NLS plasmid. UD of the *Cas9* mRNA sequence was performed with the “optimize codons” tool in Geneious version R8.1.8 (https://www.geneious.com).^68^ A new sequence in Geneious was created for the Cas9 ORF; this sequence was selected, and under the tab “annotate and predict,” the “optimize codons” function was chosen. Parameters were chosen as follows: source of genetic code, standard; target organism, *Homo sapiens*; target genetic code, standard; threshold to be rare = 1; and avoid restriction sites, No. Base content for our standard Cas9 ORF was as follows: 28.6% A, 27.8% C, 28.1% G, 15.5% U, and 55.8% GC. Base content for our uridine-depleted Cas9 ORF was as follows: 25.3% A, 30.6% C, 31.5% G, 12.6% U, and 62.1% GC.

### *In Vitro* Transcription of Modified mRNAs

Chemically modified, co-transcriptionally capped Cap 1 Cas9 and firefly luciferase (FLuc) mRNAs were synthesized by T7 RNA polymerase *in vitro* transcription. All enzymes were purchased from New England Biolabs (Ipswich, MA). Transcriptions were done in 1× transcription buffer (40 mM Tris, 10 mM dithiothreitol, 2 mM spermidine, 0.002% Triton X-100, and 27 mM magnesium acetate) using final concentrations of 8 U/μL T7 RNA polymerase (M0251L); 0.002 U/μL inorganic pyrophosphatase (M2403L); 1 U/μL murine RNase inhibitor (M0314L); 0.025 μg/μL standard or uridine-depleted transcription template; 5 mM CleanCap Cap 1 AG trimer; and 5 mM each of ATP, cytidine triphosphate (CTP) (or CTP analog), GTP, and uridine triphosphate (UTP) (or UTP analog), as indicated in Table 1. Transcription reactions were incubated at 37°C for 2 hr and treated with final 0.4 U/μL DNase I (M0303L) in 1× DNase I buffer for 15 min at 37°C. We initially made anti-reverse CleanCap trimers with a 3′-O-methyl group on the sugar of the ^m7^G to prevent incorporation in the opposite orientation, but we found this to be unnecessary, as the 3′-O-methyl version functioned equivalently to CleanCap with a 3′ OH. mRNAs were purified by RNeasy Maxi (QIAGEN, 75162), phosphatase treated for 1 hr with final 0.25 U/μg Antarctic phosphatase (M0289L) in 1× Antarctic phosphatase buffer, and then re-purified by RNeasy. A portion of each mRNA was purified by HPLC as described by Kariko et al.,^60^ except that mRNA was recovered from HPLC fractions by RNeasy purification. Purification was carried out on a PRP-H1 column (Hamilton Company) at 65°C using a gradient of 100 mM triethylammonium acetate/acetonitrile. Transcription quality was measured by bioanalyzer analysis (Agilent 2100 Bioanalyzer). mRNA concentrations were measured by UV spectroscopy and corrected for predicted extinction coefficient.

### dsRNA Slot Blot

Detection of dsRNA was performed by slot blot based on previously established methods^60^ adapted for use with a 48-well slot blot vacuum manifold (GE Whatman, Pittsburgh, PA, 10447941) and SNAP i.d. 2.0 Protein Detection System (EMD Millipore, Burlington, MA, SNAP2MIDI). In brief, RNA samples (1,000 or 200 ng) were blotted on a super-charged nytran membrane (GE Amersham, Pittsburgh, PA, 10416230) pre-wetted in 1× TBST (50 mM Tris-HCL, 138 mM NaCl, 27 mM KCl, and 0.05% Tween-20 [pH 7.5]) by applying vacuum. The membrane was then transferred to a SNAP i.d. apparatus and blocked with 30 mL 0.5% w/v nonfat dried milk in 1× TBST for 1 min prior to the application of vacuum. Blocking buffer was incubated over the membrane for 1 min before the vacuum was applied. The membrane was probed with 15 mL 1:1,500 dsRNA-specific monoclonal antibody (mAb) J2 (English & Scientific Consulting, Hungary) in 0.5% milk for 20 min and washed 4 times with 30 mL 1× TBST. The membrane was incubated for 20 min with 15 mL 1:1,500 horseradish peroxidase (HRP)-conjugated donkey anti-mouse immunoglobulin G (IgG) (Jackson ImmunoResearch, West Grove, PA, 715-035-150) in 0.5% milk and washed 4 times with 1× TBST. The membrane was developed in the dark with 30 mL enhanced chemiluminescence (ECL) western blotting detection reagent (GE Amersham, RPN2134) for 5 min before being imaged on G:BOX Chemi XRQ (Syngene, Frederick, MD) chemiluminescent imaging system with accompanying GeneSys (version [v.]1.5.6) software. Raw light units (RLUs) were measured and background corrected using densitometry software (GeneSys). For comparison of HPLC samples, RLU signals per mRNA were normalized to matched non-HPLC and reported as a percentage. Similarly, for comparison of UD samples, RLU signals per mRNA were normalized to non-UD and reported as a percentage. Percent reduction in dsRNA signal resulting from HPLC purification or UD was calculated using the following formula:

$$\%\;\;\text{reduction}=100-\left(\frac{\text{HPLC intensity}}{\text{non}-\text{HPLC intensity}}\right)*100$$

$$\%\;\text{reduction}=100-\left(\frac{\text{uridine}-\text{depleted intensity}}{\text{WT intensity}}\right)*100.$$

We calculated this for the 200- and 1,000-ng inputs and averaged these two values.

### THP-1 and THP-1 Dual Cell Culture

THP-1 cells (ATCC, TIB-202) and THP-1 Dual cells (InvivoGen, San Diego, CA, thpd-nfis) were grown in RPMI-1640 medium (ATCC, 30-2001) supplemented with 10% fetal bovine serum (Gibco, Grand Island, NY, 10437-028), 1 mM sodium pyruvate (Gibco, 11360-070), 1× MEM non-essential amino acids (Gibco, 11140-050), 100 U/mL penicillin and 100 μg/mL streptomycin (Gibco, 15140-122), and 100 μg/mL Normocin (InvivoGen, ant-nr-1) at 37°C in an atmosphere of 5% CO_2_. THP-1 Dual cells were grown in the presence of 100 μg/mL zeocin (InvivoGen, ant-zn-1) and 10 μg/mL blasticidin (InvivoGen, ant-bl-1) every other passage to maintain positive selection of reporters.

### Luciferase Assay in Cultured THP-1 Cells

In preparation for THP-1 cell transfections, 2 × 10^5^ cells per well were seeded in a 24-well plate format (Corning Costar, Tewksberry, MA, 3527) and allowed to differentiate in culture for 72 hr using 200 nM phorbol ester 12-O-Tetradecanoylphorbol-13-Acetate (Cell Signaling Technology, Danvers, MA, 4174). Cells were then transfected with 100 ng *in vitro*-transcribed, non-HPLC, and HPLC-purified unmodified or modified FLuc mRNAs complexed with 1 μL transfection reagent mRNA-In (MTI-GlobalStem, Gaithersburg, MA, 73741) and Opti-MEM (Gibco, 11058-021) in a total volume of 50 μL. Complexed mRNAs were briefly vortexed and incubated for 10 min at room temperature, then added drop-wise to each well. Modified FLuc mRNAs were transfected in sextuplicate. 24 hr post-transfection, media were aspirated from each transfected well, and adhered monolayers were lysed with the ONE-Glo Luciferase Assay System (Promega, Madison, WI, E6120) to assay for FLuc activity. Lysates were incubated in the dark for 10 min at room temperature with gentle rocking, then transferred to a white 96-well microtiter plate (Greiner Bio-One, Monroe, NC, 655073). Luciferase activity was measured using a GloMax Multi+ Detection System luminometer (Promega, E8032) with a 0.5-s integration per well.

### IFN Response Assay in Cultured THP-1 Cells

THP-1 Dual cells were seeded, differentiated, and transfected as above except that cells were transfected with 100 ng *in vitro*-transcribed, non-HPLC, and HPLC-purified unmodified or modified *Cas9* mRNAs. Modified *Cas9* mRNAs were transfected in sextuplicate, and supernatants from each transfected well were assayed for Lucia activity 24 hr post-transfection. To assay Lucia activity as a measure of an IFN response, 50 μL media were mixed with 150 μL QUANTI-Luc coelenterazine luciferase substrate (InvivoGen, rep-qlc) in a white 96-well microplate (Greiner Bio-One, 655073), and luminescence was measured using a GloMax Multi+ Detection System luminometer (Promega, E8032) with a 10-s integration per well.

### CD34^+^ HSPC Tissue Culture

CD34^+^ HSPCs derived from mobilized peripheral blood donated by male donors were purchased from AllCells (Alemeda, CA). Cells were thawed according to the manufacturer’s instructions and cultured at a density of 250,000/mL in a 24-well plate. CD34^+^ HSPCs were cultured in StemSpan SFEM II (STEMCELL Technologies, Vancouver, Canada) supplemented with stem cell factor (100 ng/mL), thrombopoietin (100 ng/mL), Flt3-Ligand (100 ng/mL), IL-6 (100 ng/mL), StemRegenin1 (0.75 mM), and UM171 (STEMCELL Technologies, 35 nM). Cells were cultured at 37°C, 5% CO_2_, and 5% O_2_.

### Nucleofection of CD34+ HSPCs

Nucleofection was performed 48 hr after cells were thawed. Cell viability was confirmed to be >80% using trypan blue before nucleofection. Cells were resuspended in 1 M buffer (5 mM KCl, 15 mM MgCl_2_, 120 mM Na_2_HPO_4_/NaH_2_PO_4_ [pH7.2], and 50 mM Manitol) at a density of 5 million cells/mL. As a control, we also included Cas9 RNP at a Cas9:sgRNA molar ratio of 1:2.5 as previously described by Hendel et al.^6^ Briefly, 6 μg Cas9 protein was incubated with 3.2 μg IL2RG locus MS-sgRNA^6^ (ACAACTTCGGTAGTAATGGT…) for 15 min prior to nucleofection. 3 μg *Cas9* mRNA and 2 μg MS-sgRNA were used for nucleofections.^6^, ^69^ *Cas9* mRNA and RNP were then mixed with 100,000 CD34^+^ cells (20 μL of cell suspension) and transferred to a 16-well nucleofection strip (Lonza, MD, USA). Each treatment was performed in duplicate. Cells were nucleofected using DZ100 program in the Lonza 4D nucleofector. Cells were suspended in 200 μL CD34^+^ media after nucleofection.

### Measurement of Indels

Genomic DNA was obtained using QuickExtract DNA Extraction Solution (Epicenter, Madison, WI). The mixture was vortexed and incubated at 65°C for 6 min followed by 100°C for 10 min, a slight deviation from the manufacturer’s recommendations for more optimal downstream applications. The target sequence in the IL2RG locus was amplified using PCR and sequenced. Indels were measured using TIDE software as previously described.^61^ Briefly, the software uses quantitative sequence trace data from control cells and cells edited using Cas9 RNP or mRNA. The software decomposes the edited sequence trace into individual components using multi-variate non-negative linear modeling, and it uses the control sequence as a template to model indels.

The following primers were used for PCR amplification of the IL2RG site: forward, 5′-TCACACAGCACATATTTGCCACACCCT-3′ and reverse, 5′-TGCCCACATGATTGTAATGGCCAGTGG-3′.

### Whole-Blood Assay

Blood samples from three different healthy donors was taken and collected in EDTA collection tubes (Sarstedt, Germany). For each treatment group, 2 mL EDTA-blood was transferred into 12-well plates and treated accordingly. 10 μL 1 mg/mL (un-)modified mRNAs were complexed to 10 μL TransIT (Mirus Bio, Madison, WI). For a positive control group, blood was treated with the TLR 7 and 8 agonist R-848 (Resiquimod, Sigma-Aldrich, St. Louis, MO). Samples were incubated for 6 or 24 hr at 37°C in a humidified atmosphere containing 5% CO_2_. At each time point, 1 mL whole blood was transferred into columns for serum separation (Sarstedt, 41.1378.005) and spun down at 10,000 × *g* for 5 min to obtain serum. Sera were stored at −20°C until further cytokine measurement analyses.

### Animal Experiments

All animal experiments were approved by the local ethics committee and carried out according to the guidelines of the German Law for the Protection of Animals. BALB/cJ mice were purchased from Jackson Laboratory (Bar Harbor, ME) at an age of 6–8 weeks and were maintained under standardized specific-pathogen-free conditions on a 12-hr light-dark cycle. Nesting material was provided and food and water were provided *ad libitum.* Prior to injections, mice were anesthetized intraperitoneally with a mixture of medetomidine (0.5 mg/kg), midazolam (5 mg/kg), and fentanyl (50 μg/kg). BALB/cJ mice received 20 μg *Cas9* mRNA encapsulated in chitosan-coated PLGA nanoparticles (Chitosan, 83% deacetylated [Protasan UP CL 113] coated PLGA 75:25 [Resomer RG 752H] nanoparticles [NPs]) by intravenous injection (n = 3) into the tail vein. For both interventions, mRNA-NPs were administered in a total volume of 200 μL. To assess immune responses after 6 and 24 hr, mice were sacrificed and blood was collected to obtain serum.

### Cytokine Measurement

Blood from mice and human donors was used to obtain serum and tested for IL-12, IL-6, and TNF-α (human and mice, respectively) production by ELISA, as directed in the manufacturer’s instructions (BD Biosciences, San Jose, CA).

### Statistics

Data were analyzed using Prism 6 Software (GraphPad, San Diego, CA) using a 95% confidence interval. For indel measurements, data were analyzed by one-way ANOVA with a Tukey’s multiple comparisons test. For IFN, mouse cytokine, and whole-blood cytokine measurements, an ANOVA with a Dunnet’s test for multiple comparisons was used.

## Author Contributions

S.V., K.T.A., A.K.M.A.H., J.M.H., A.H., S.S., J.S.A., R.I.H., M.S.D.K., M.H.P., and A.P.M. designed experiments. S.V., K.T.A., A.K.M.A.H., J.M.H., A.H., S.S., and J.S.A. conducted experiments. S.V., K.T.A., A.K.M.A.H., J.M.H., S.S., J.S.A., and A.P.M. wrote the manuscript.

## Conflicts of Interest

M.H.P. receives consulting fees from CRISPR Therapeutics for serving on their scientific advisory board.

## Supplemental Information

Document S1. Figures S1–S5

Document S2. Article plus Supplemental Information
