# Supplementary material for: Uridine Depletion and Chemical Modification Increase Cas9 mRNA Activity and Reduce Immunogenicity without HPLC Purification
Source: Mol Ther Nucleic Acids. 2018 Jun 30;12:530–42. doi: 10.1016/j.omtn.2018.06.010 (PMC6076213; doi:10.1016/j.omtn.2018.06.010)
Supplement: Document S1. Figures S1–S5 [file mmc1.pdf]

OMTN, Volume *12*

Supplemental Information

Uridine Depletion and Chemical Modification Increase *Cas9* mRNA Activity and Reduce Immunogenicity without HPLC Purification

Sriram Vaidyanathan, Krist T. Azizian, A.K.M. Ashiqul Haque, Jordana M. Henderson, Ayal Hendel, Sabrina Shore, Justin S. Antony, Richard I. Hogrefe, Michael S.D. Kormann, Matthew H. Porteus, and Anton P. McCaffrey

Supplemental Figures and Legends

Supplemental Fig. 1

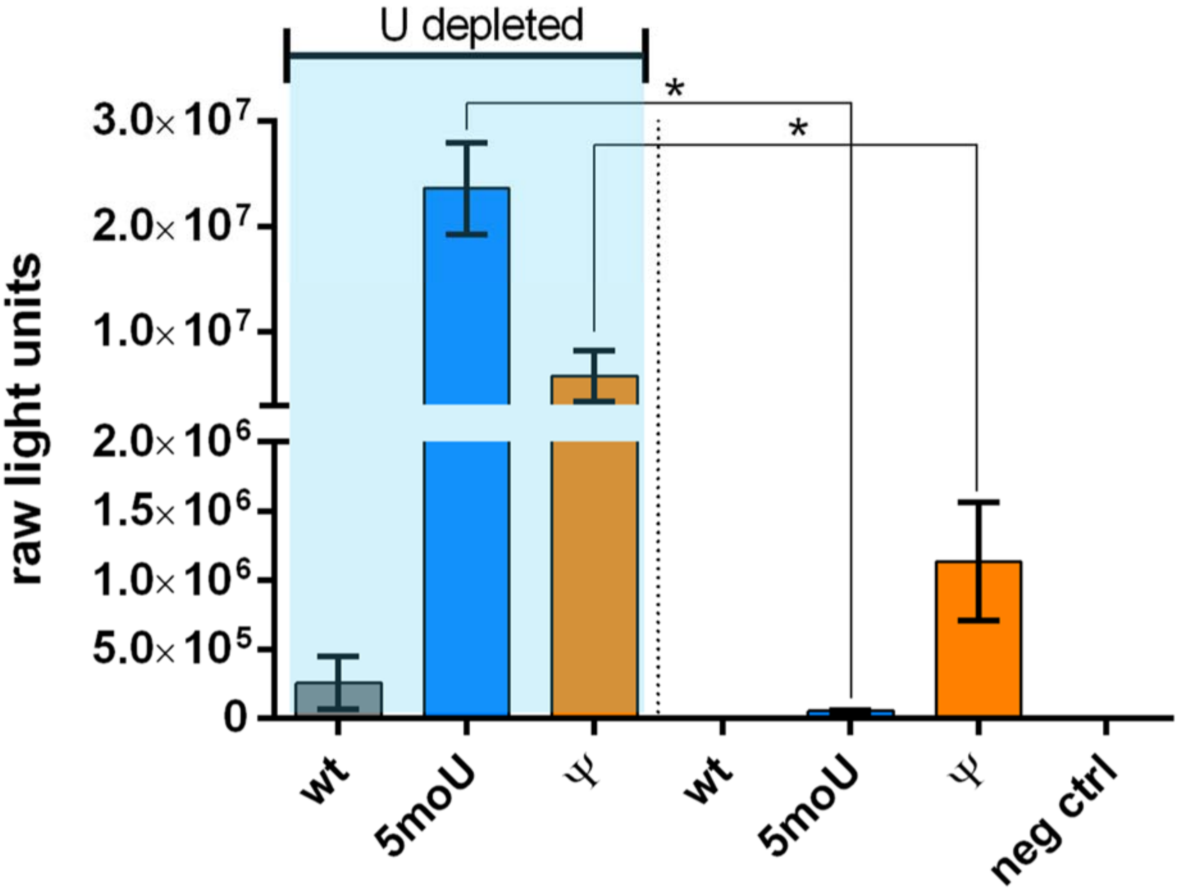

Supplemental Figure 1. Luciferase activity from transfected mRNAs in THP-1 cells

Cells were transfected with 100 ng of FLuc mRNA complexed with 1 µl of mRNA-In transfection reagent in sextuplicate. At 24 hours, cells were lysed and Luciferase activity was measured. Bars represent mean +/- standard error of the mean (SEM) of two independent assays comprising a total of twelve replicates. * indicates $p<0.05$.

Supplemental Fig. 2

Supplemental Fig. 2A

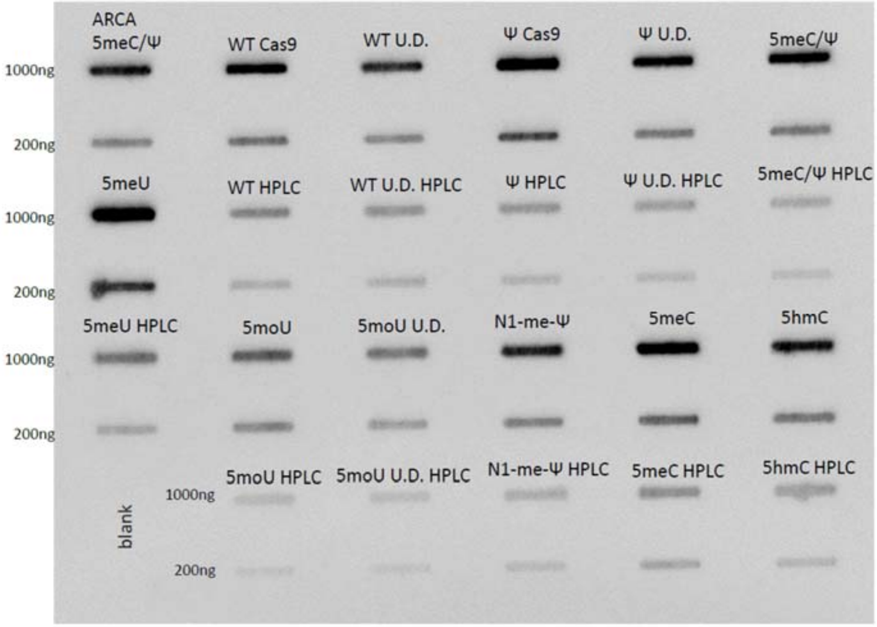

Supplemental Fig. 2B

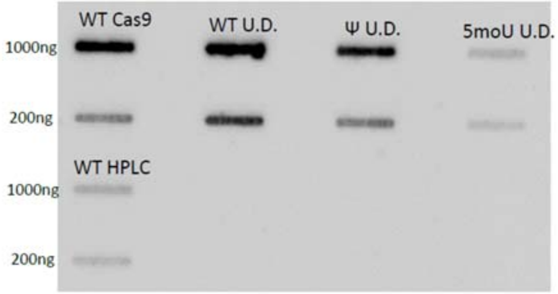

Supplemental Fig. 2C

Relative Signal Reduction

WT (HPLC)	65.8%
WT U.D. (HPLC)	54.0%
Ψ (HPLC)	80.3%
Ψ U.D. (HPLC)	71.6%
5meC/Ψ (HPLC)	76.5%
5meU (HPLC)	77.3%
5moU (HPLC)	76.9%
5moU U.D. (HPLC)	71.0%
N1-me-Ψ (HPLC)	72.5%
5meC (HPLC)	71.1%
5hmC (HPLC)	70.4%

Supplemental Fig. 2D

WT (U.D.)	32.1%
5moU (U.D.)	29.1%
Ψ (U.D.)	39.3%

Supplemental Figure 2. Slot blot of non-HPLC and HPLC-purified modified *Cas9* mRNAs

To measure depletion of dsRNA upon HPLC purification, a previously published dot blot method [60] was adapted to slot blot to improve quantitation. Either 200 ng or 1000 ng was loaded. Blot was probed with a J2 antibody specific for dsRNA and developed with ECL reagent. A) Original syntheses of mRNAs. B) A second synthesis of selected mRNAs was completed after original reagents from A were depleted. C) The average relative reduction in dsRNA after HPLC purification quantified from the blot in A. dsRNA depletion was estimated by comparing intensities

of raw light units (RLU) using densitometry software. RLU signlals per mRNA were normalized to matched non-HPLC and reported as the average percentage between 200ng and 1000ng inputs. D) Similarily, the relative dsRNA signal reduction between U.D samples and non-U.D samples of WT, 5moU and Ψ from blot in A.

Supplemental Fig. 3

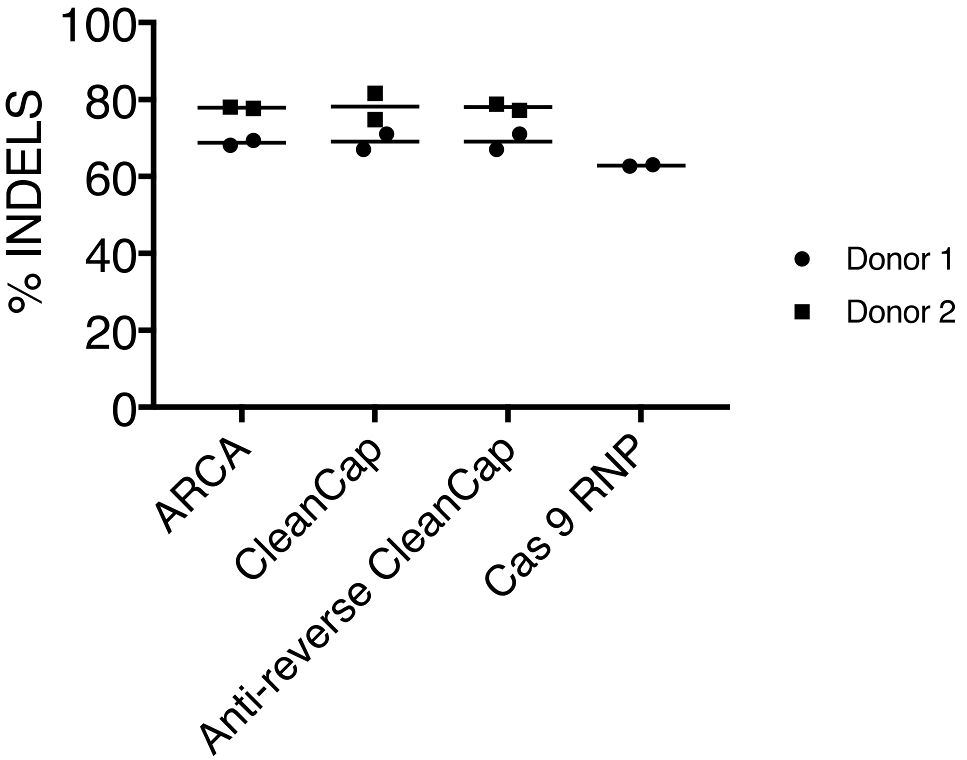

Supplemental Figure 3. Indel activity of 5moU U.D with Cap 0 and Cap 1 structures

CD34+ cells were electroporated with cas9 mRNA with Cap 0 and Cap 1 structures and IL2RG MS-sgRNA and indel activity was measured on day 4. There was no significant difference between groups as determined by one-way ANOVA. (p=0.14)

Supplemental Fig. 4

Supplemental Fig. 4a

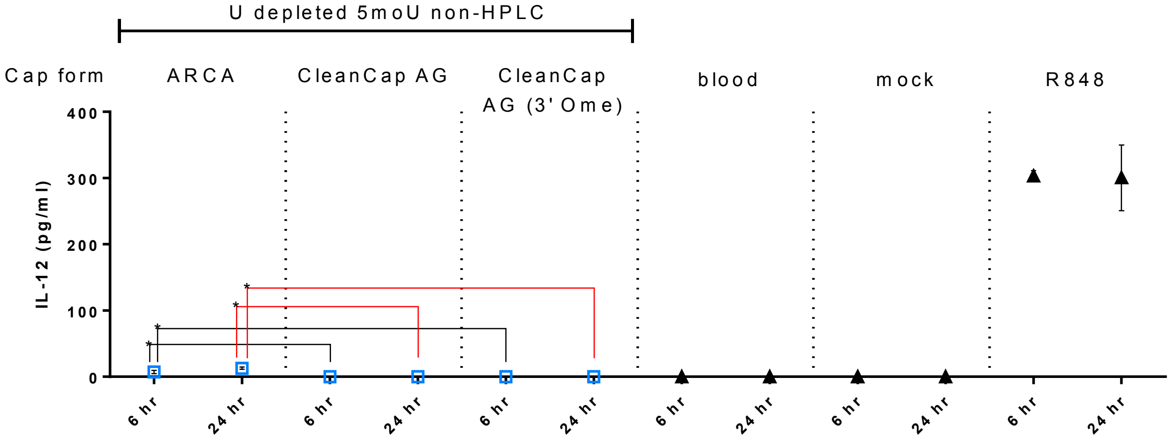

Supplemental Fig. 4b

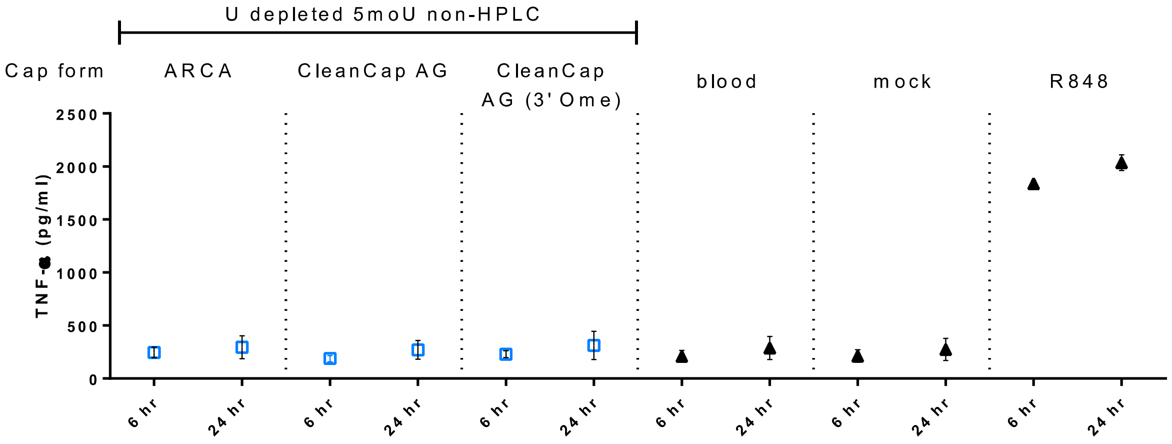

Supplemental Fig. 4c

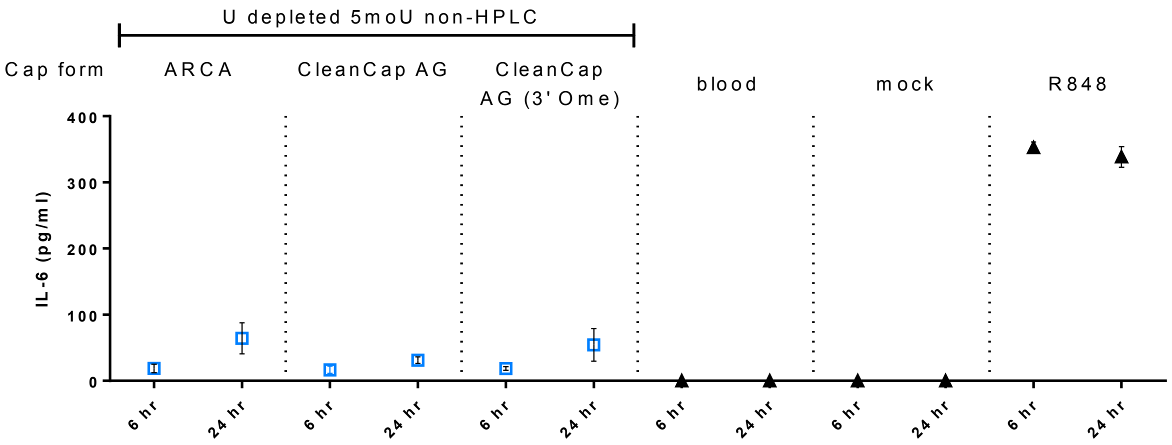

Supplemental Figure 4. Amounts of IL-12 , TNF--α and IL-6 in whole human blood transfected with uridine depleted 5moU modified *Cas9* mRNAs with different cap structures. To assess immune responses to transfected mRNAs, whole blood from healthy human volunteers (N=3) was transfected with 10 µg of mRNA complexed with 10 µl of TransIT (www.mirusbio.com). After 6 or 24 hours of incubation, sera was isolated, and A) IL-12 or B) TNF-α or C) IL-6 was measured by ELISA. Bars represent mean +/- SEM. * indicates $p<0.05$ relative to 6 hr blood-only control**.**

Supplemental Fig. 5

Supplemental Fig. 5a

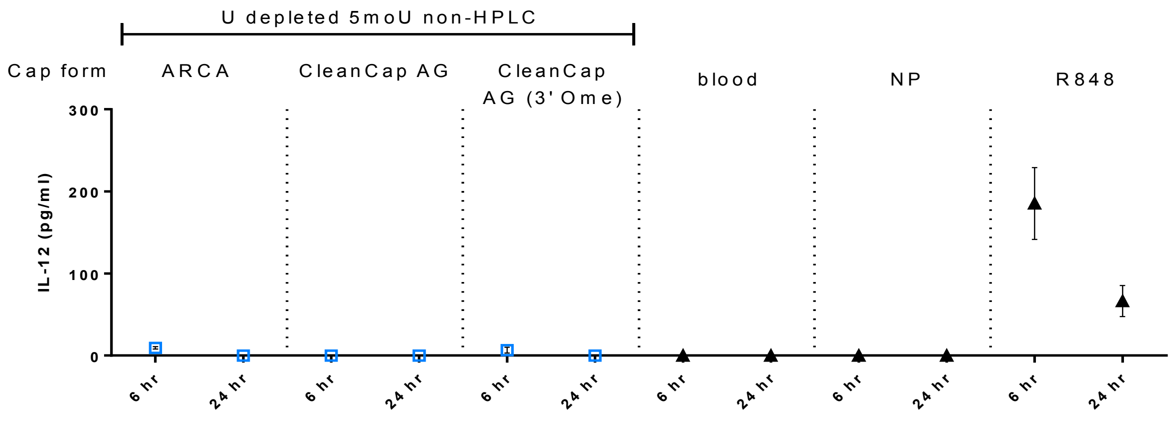

Supplemental Fig. 5b

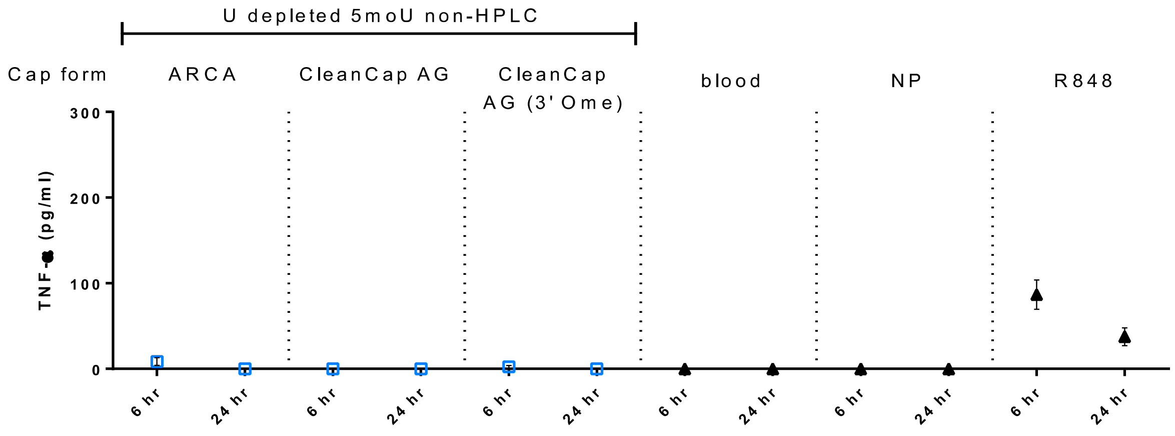

Supplemental Fig. 5c

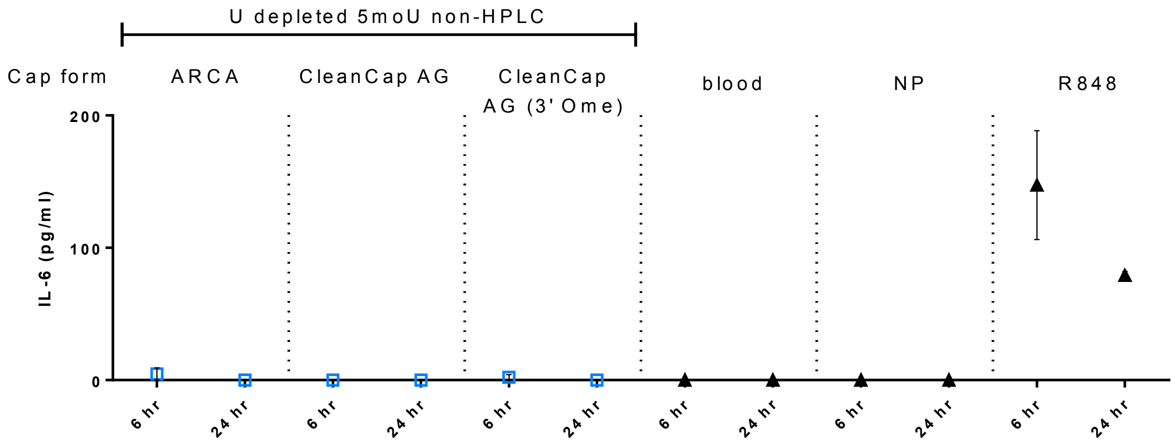

Supplemental Figure 5. Amounts of IL-12 and TNF--α in the sera of mice after IV infusion of uridine depleted 5moU modified *Cas9* mRNAs with different cap structures. To assess immune responses *in vivo*, 20 μg of *Cas9* mRNA encapsulated in chitosan-coated PLGA nanoparticles was injected intravenously (N=3) into the tail vein of mice. After 6 or 24 hours of incubation, sera was isolated, and A) IL-12 or B) TNF-α C) IL-6 was measured by ELISA. Blood treated with R-848 serves as a positive control. Bars represent mean +/- SEM. * indicates $p<0.05$ relative to 6 hr blood-only control.
